# Supplementary material for: A compartmentalized signaling network mediates crossover control in meiosis
Source: eLife. 2018 Mar 9;7:e30789. doi: 10.7554/eLife.30789 (PMC5906097; doi:10.7554/eLife.30789)
Supplement: Supplementary file 1. — Table S1 reports analysis of the progeny produced by animals expressing epitope-tagged proteins described in this work. In all cases, animals homozygous for these epitope-tagged alleles show no reduction in embryonic viability or increase in male production, indicating that the epitope tags used to detect various proteins do not impair their meiotic functions. Table S2 provides a list of all new alleles generated in this work, with details about the genome editing methods used to introduce epitope tags or mutations. Table S3 includes the sequences of RNA and DNA sequences used for genome editing, including synthetic CRISPR RNAs (crRNAs), repair templates, and DNA primers used for detecting or genotyping these mutations. In cases where restriction digests were used for genotyping, fragment sizes for wild-type and mutant alleles are also provided. Table S4 lists new worm strains constructed in the course of this work, as well as previously referenced strains. [file elife-30789-supp1.docx]

**Supplementary File 1**

**Table S1**

**Viability and fertility of representative transgenic worm strains**

| Strains | Eggs laid (±SD)  (6 Hermaphrodites) | Egg viability (±SD) (%) | Male progeny (±SD) (%) |
| --- | --- | --- | --- |
| WT | 279.83 (±35.61) | 107.62 (±5.47) | 0.16 (±0.17) |
| *zhp-1::AID::3xFLAG; GFP::cosa-1; P_sun-1_::TIR1* | 250.5 (±30.43) | 113.80 (±3.58) | 0.12 (±0.30) |
| *zhp-2::AID::3xFLAG; GFP::cosa-1; P_sun-1_::TIR1* | 270.00 (±18.25) | 110.58 (±3.27) | 0.11 (±0.17) |
| *zhp-3::AID::3xFLAG; GFP::cosa-1; P_sun-1_::TIR1* | 263.83 (±20.62) | 108.52 (±2.92) | 0.06 (±0.14) |
| *GFP::cosa-1; P_sun-1_::TIR1; zhp-4::AID::3xFLAG* | 260.33 (±16.84) | 109.12 (±2.69) | 0.34 (±0.35) |
| *zhp-2::AID::3xFLAG; GFP::cosa-1; P_sun-1_::TIR1, spo-11::AID::3xFLAG* | 277.67 (±21.90) | 110.47 (±3.21) | 0.11 (±0.17) |
| *plk-2::HA, zhp-2::AID::3xFLAG, zhp-3::V5; dsb-2::AID::3xFLAG, GFP::cosa-1; P_sun-1_::TIR1* | 270.17 (±21.10) | 108.33 (±5.31) | 0.29 (±0.35) |
| *plk-2::AID::3xFLAG, zhp-2::HA; GFP::cosa-1; P_sun-1_::TIR1* | 255.00 (±12.13) | 111.28 (±2.10) | 0.30 (±0.36) |

Note: viability of >100% is a consequence of failing to count some embryos.

**Table S2**

**Alleles generated in this study**

| Allele | Genotype | Information about mutagenesis |
| --- | --- | --- |
| *ie43* | *zhp-1(ie43)/hT2*; stop codon + frameshift + Xho1 after aa6; sequence inserted is 5’-TAAGCTCGAG-3’ | generated using *dpy-10* Co-CRISPR, balanced with *hT2 [bli-4(e937) let-?(q782) qIs48] (I; III),* #2 |
| *ie44* | *zhp-1(ie44)/hT2*; stop codon + frameshift + Xho1 after aa6; sequence inserted is 5’-TAAGCTCGAG-3’ | generated using *dpy-10* Co-CRISPR, balanced with *hT2 [bli-4(e937) let-?(q782) qIs48] (I; III)*, #4 |
| *ie45* | *zhp-2(ie45)/hT2*; stop codon + frameshift after aa7; nucleotide c22 was replaced by 5’-TAATAATTAATTAG-3’ | generated using *dpy-10* Co-CRISPR, balanced with *hT2 [bli-4(e937) let-?(q782) qIs48] (I; III)*, #18 |
| *ie46* | *zhp-1(ie46)*; stop codon + frameshift + Xho1 after aa6; sequence inserted is 5’-TAAGCTCGAG-3’; in Hawaii CB4586 background | generated using *dpy-10* Co-CRISPR in Hawaii CB4586. #1 |
| *ie51* | *htp-3(ie51)* mutant | generated using *dpy-10* Co-CRISPR in *zhp-1(ie62[zhp-1::AID::3xFLAG]) I; meIs8[pie-1p::GFP::cosa-1, unc-119(+)] II; ieSi38[sun-1p::TIR1::mRuby::sun-1 3'UTR, Cbr-unc-119(+)] IV* |
| *ie52* | *htp-3(ie52)* mutant | generated using *dpy-10* Co-CRISPR in *zhp-2(ie67[zhp-2::AID::3xFLAG]) I; meIs8[pie-1p::GFP::cosa-1, unc-119(+)] II; ieSi38[sun-1p::TIR1::mRuby::sun-1 3'UTR, Cbr-unc-119(+)] IV* |
| *ie58* | *dsb-2(ie58[dsb-2::AID::3xFLAG])* | generated using *dpy-10* Co-CRISPR in *zhp-2(ie67[zhp-2::AID::3xFLAG]) I; meIs8[pie-1p::GFP::cosa-1, unc-119(+)] II; ieSi38[sun-1p::TIR1::mRuby::sun-1 3'UTR, Cbr-unc-119(+)] IV* |
| *ie59* | *spo-11(ie59[spo-11::AID::3xFLAG])* | generated using *dpy-10* Co-CRISPR in *zhp-1(ie62[zhp-1::AID::3xFLAG]) I; meIs8[pie-1p::GFP::cosa-1, unc-119(+)] II; ieSi38[sun-1p::TIR1::mRuby::sun-1 3'UTR, Cbr-unc-119(+)] IV* |
| *ie60* | *spo-11(ie60[spo-11::AID::3xFLAG])* | generated using *dpy-10* Co-CRISPR in *zhp-2(ie67[zhp-2::AID::3xFLAG]) I; meIs8[pie-1p::GFP::cosa-1, unc-119(+)] II; ieSi38[sun-1p::TIR1::mRuby::sun-1 3'UTR, Cbr-unc-119(+)] IV* |
| *ie61* | *zhp-1(ie61[zhp-1::AID::3xFLAG])* | generated using *dpy-10* Co-CRISPR in *ieSi38[sun-1p::TIR1::mRuby::sun-1 3'UTR, Cbr-unc-119(+)] IV* |
| *ie62* | *zhp-1(ie62[zhp-1::AID::3xFLAG])* | generated using *dpy-10* Co-CRISPR in *meIs8[pie-1p::GFP::cosa-1, unc-119(+)] II; ieSi38[sun-1p::TIR1::mRuby::sun-1 3'UTR, Cbr-unc-119(+)] IV* |
| *ie63* | *zhp-2(ie63[zhp-2::HA])* | generated using *dpy-10* Co-CRISPR in *zhp-1(ie61[zhp-1::AID::3xFLAG]) I; ieSi38[sun-1p::TIR1::mRuby::sun-1 3'UTR, Cbr-unc-119(+)] IV* |
| *ie64* | *zhp-3(ie64[zhp-3::V5])* | generated using *dpy-10* Co-CRISPR in *zhp-1(ie61[zhp-1::AID::3xFLAG]) I; ieSi38[sun-1p::TIR1::mRuby::sun-1 3'UTR, Cbr-unc-119(+)] IV* |
| *ie65* | *zhp-4(ie65[zhp-4::HA])* | generated using *dpy-10* Co-CRISPR in *zhp-1(ie61[zhp-1::AID::3xFLAG]) I; ieSi38[sun-1p::TIR1::mRuby::sun-1 3'UTR, Cbr-unc-119(+)] IV* |
| *ie66* | *zhp-2(ie66[zhp-2::AID::3xFLAG])* | generated using *dpy-10* Co-CRISPR in *ieSi38[sun-1p::TIR1::mRuby::sun-1 3'UTR, Cbr-unc-119(+)] IV* |
| *ie67* | *zhp-2(ie67[zhp-2::AID::3xFLAG])* | generated using *dpy-10* Co-CRISPR in *meIs8[pie-1p::GFP::cosa-1, unc-119(+)] II; ieSi38[sun-1p::TIR1::mRuby::sun-1 3'UTR, Cbr-unc-119(+)] IV* |
| *ie68* | *zhp-1(ie68[zhp-1::V5])* | generated using *dpy-10* Co-CRISPR in *zhp-2(ie66[zhp-2::AID::3xFLAG]) I; ieSi38[sun-1p::TIR1::mRuby::sun-1 3'UTR, Cbr-unc-119(+)] IV* |
| *ie69* | *zhp-3(ie69[zhp-3::V5])* | generated using *dpy-10* Co-CRISPR in *zhp-2(ie66[zhp-2::AID::3xFLAG]) I; ieSi38[sun-1p::TIR1::mRuby::sun-1 3'UTR, Cbr-unc-119(+)] IV* |
| *ie70* | *zhp-4(ie70[zhp-4::HA])* | generated using *dpy-10* Co-CRISPR in *zhp-2(ie66[zhp-2::AID::3xFLAG]) I; ieSi38[sun-1p::TIR1::mRuby::sun-1 3'UTR, Cbr-unc-119(+)] IV* |
| *ie72* | *zhp-2(ie72[zhp-2::HA])* | generated using Cas9 in *syp-1(me17) V/nT1[unc-?(n754) let-? qIs50] (IV;V)* |
| *ie73* | *zhp-2(ie73[zhp-2::HA])* | generated using *dpy-10* Co-CRISPR in *spo-11(me44)/ieT1* |
| *ie74* | *zhp-2(ie74[zhp-2::HA])* | generated using CRISPR/Cas9 in *msh-5(me23) IV/nT1[unc-?(n754) let-?] (IV;V)* |
| *ie75* | *zhp-2(ie75[zhp-2::HA])* | generated using CRISPR/Cas9 in *cosa-1(we12) III/qC1 III* |
| *ie76* | *zhp-3(ie76[zhp-3::AID::3xFLAG])* | generated using *dpy-10* Co-CRISPR in *ieSi38[sun-1p::TIR1::mRuby::sun-1 3'UTR, Cbr-unc-119(+)] IV* |
| *ie77* | *zhp-3(ie77[zhp-3::AID::3xFLAG])* | generated using *dpy-10* Co-CRISPR in *meIs8[pie-1p::GFP::cosa-1, unc-119(+)] II; ieSi38[sun-1p::TIR1::mRuby::sun-1 3'UTR, Cbr-unc-119(+)] IV* |
| *ie78* | *zhp-1(ie78[zhp-1::V5])* | generated using *dpy-10* Co-CRISPR in *zhp-3(ie76[zhp-3::AID::3xFLAG]) I; ieSi38[sun-1p::TIR1::mRuby::sun-1 3'UTR, Cbr-unc-119(+)] IV* |
| *ie79* | *zhp-2(ie79[zhp-2::HA])* | generated using *dpy-10* Co-CRISPR in *zhp-3(ie76[zhp-3::AID::3xFLAG]) I; ieSi38[sun-1p::TIR1::mRuby::sun-1 3'UTR, Cbr-unc-119(+)] IV* |
| *ie80* | *zhp-3(ie80[zhp-3::AID::GFP])* | generated using *dpy-10* Co-CRISPR in *ieSi38[sun-1p::TIR1::mRuby::sun-1 3'UTR, Cbr-unc-119(+)] IV* |
| *ie81* | *zhp-4(ie81[zhp-4::HA])* | generated using *dpy-10* Co-CRISPR in *zhp-3(ie80[zhp-3::AID::GFP]) I; ieSi38[sun-1p::TIR1::mRuby::sun-1 3'UTR, Cbr-unc-119(+)] IV* |
| *ie82* | *zhp-4(ie82[zhp-4::AID::3xFLAG])* | generated using *dpy-10* Co-CRISPR in *ieSi38[sun-1p::TIR1::mRuby::sun-1 3'UTR, Cbr-unc-119(+)] IV* |
| *ie83* | *zhp-1(ie83[zhp-1::V5])* | generated using *dpy-10* Co-CRISPR in *ieSi38[sun-1p::TIR1::mRuby::sun-1 3'UTR, Cbr-unc-119(+)] IV; zhp-4(ie82[zhp-4::AID::3xFLAG]) V* |
| *ie84* | *zhp-2(ie84[zhp-2::HA])* | generated using *dpy-10* Co-CRISPR in *ieSi38[sun-1p::TIR1::mRuby::sun-1 3'UTR, Cbr-unc-119(+)] IV; zhp-4(ie82[zhp-4::AID::3xFLAG]) V* |
| *ie85* | *zhp-3(ie85[zhp-3::V5])* | generated using *dpy-10* Co-CRISPR in *ieSi38[sun-1p::TIR1::mRuby::sun-1 3'UTR, Cbr-unc-119(+)] IV; zhp-4(ie82[zhp-4::AID::3xFLAG]) V* |
| *ie86* | *zhp-4(ie86[zhp-4::HA])* | generated using *dpy-10* Co-CRISPR in *zhp-3(jf61)/hT2 [bli-4(e937) let-?(q782) qIs48] (I; III)* |
| *ie87* | *zhp-4(ie87[zhp-4::HA])* | generated using CRISPR/Cas9 in *syp-1(me17) V/nT1[unc-?(n754) let-? qIs50] (IV;V)* |
| *ie88* | *zhp-4(ie88[zhp-4::HA])* | generated using *dpy-10* Co-CRISPR in *spo-11(me44)/ieT1* |
| *ie89* | *zhp-4(ie89[zhp-4::HA])* | generated using CRISPR/Cas9 in *msh-5(me23) IV/nT1[unc-?(n754) let-?] (IV;V)* |
| *ie90* | *zhp-4(ie90[zhp-4::HA])* | generated using CRISPR/Cas9 in *cosa-1(we12) III/qC1 III* |
| *ie92* | *zhp-3(ie92[zhp-3::V5])* | generated using *dpy-10* Co-CRISPR |
| *ie103* | *zhp-3(ie103[zhp-3::V5])* | generated using *dpy-10* Co-CRISPR in *dsb-2(ie58[dsb-2::AID::3xFLAG]), meIs8[pie-1p::GFP::cosa-1, unc-119(+)] II; ieSi38[sun-1p::TIR1::mRuby::sun-1 3'UTR, Cbr-unc-119(+)] IV* |
| *ie104* | *zhp-3(ie104[zhp-3::V5])* | generated using *dpy-10* Co-CRISPR in *zhp-2(ie67[zhp-2::AID::3xFLAG]) I; dsb-2(ie58[dsb-2::AID::3xFLAG]), meIs8[pie-1p::GFP::cosa-1, unc-119(+)] II; ieSi38[sun-1p::TIR1::mRuby::sun-1 3'UTR, Cbr-unc-119(+)] IV* |
| *ie105* | *plk-2(ie105[plk-2::HA])* | generated using *dpy-10* Co-CRISPR in *zhp-3(ie103[zhp-3::V5]) I; dsb-2(ie58[dsb-2::AID::3xFLAG]), meIs8[pie-1p::GFP::cosa-1, unc-119(+)] II; ieSi38[sun-1p::TIR1::mRuby::sun-1 3'UTR, Cbr-unc-119(+)] IV* |
| *ie106* | *plk-2(ie106[plk-2::HA])* | generated using *dpy-10* Co-CRISPR in *zhp-2(ie67[zhp-2::AID::3xFLAG]), zhp-3(ie104[zhp-3::V5]) I; dsb-2(ie58[dsb-2::AID::3xFLAG]), meIs8[pie-1p::GFP::cosa-1, unc-119(+)] II; ieSi38[sun-1p::TIR1::mRuby::sun-1 3'UTR, Cbr-unc-119(+)] IV* |
| *ie107* | *zhp-2(ie107[zhp-2::HA])* | generated using *dpy-10* Co-CRISPR in *meIs8[pie-1p::GFP::cosa-1, unc-119(+)] II; ieSi38[sun-1p::TIR1::mRuby::sun-1 3'UTR, Cbr-unc-119(+)] IV*. (*ie107* and *ie108* were made from a single injection) |
| *ie108* | *plk-2(ie108[plk-2::AID::3xFLAG])* | generated using *dpy-10* Co-CRISPR in *meIs8[pie-1p::GFP::cosa-1, unc-119(+)] II; ieSi38[sun-1p::TIR1::mRuby::sun-1 3'UTR, Cbr-unc-119(+)] IV*. (*ie107* and *ie108* were made from a single injection) |
| *ie109* | *plk-2(ie109[plk-2::HA])* | generated using *dpy-10* Co-CRISPR in *zhp-1(ie62[zhp-1::AID::3xFLAG]) I; meIs8[pie-1p::GFP::cosa-1, unc-119(+)] II; ieSi38[sun-1p::TIR1::mRuby::sun-1 3'UTR, Cbr-unc-119(+)] IV* |
| *ie110* | *plk-2(ie110[plk-2::HA])* | generated using *dpy-10* Co-CRISPR in *zhp-2(ie67[zhp-2::AID::3xFLAG]) I; meIs8[pie-1p::GFP::cosa-1, unc-119(+)] II; ieSi38[sun-1p::TIR1::mRuby::sun-1 3'UTR, Cbr-unc-119(+)] IV* |

**Table S3**

**crRNAs, repair templates and genotyping primers for transgenes generated in this study**

| Transgenes | crRNAs and repair templates | Genotyping primers and fragment sizes |
| --- | --- | --- |
| *zhp-1* null mutant in *ie43* or *ie44* | 5’- ttcatcgtttgcaatggatg -3’; 5’-gttgttcggcatgtttcacagaagacgtgggagcaggcggtgataaaaaattgtcttttacttggcgagcatccacatccattctcgagcttagcaaacgatgaactccatagtgcctacagttctgaaataaatgcaaattgttatttttgaaccatgtttggaacg -3’ | F: 5’- gaaaattatcgaaaaactgtcacaaac -3’; R: 5’- gttttccattttcaattgaattgacttc -3’;  WT, 563bp; Mutant, 573bp=297bp+276bp (XhoI digestion) |
| *zhp-2* null mutant in *ie45* | 5’- tggatccaatgcaatcattg -3’; 5’-ctttcgtacagttttgacagaaaacgtgaccgcatgctgttaggtacagtttcgtttgatttggtttaatgccacaatgattgcctcgagttagcattggatccaatccatacgtgtttcgagtcttaaaatacgattaaataaagctcaaagacttctgctagataacaag -3’ | Nucleotide c22 was replaced by taataattaattag.  F: 5’ gactcgaaacacgtatggattg-3’;  R: 5’-ggtacagtttcgtttgatttg-3’;  WT, 72bp; Mutant, 85bp |
| *zhp-1* null mutant in *ie46* | 5’- ttcatcgtttgcaatggatg -3’; 5’- gttgttcggcatgtttcacagaagacgtgggagcaggcggtgataaaaaattgtcttttacttggcgagcatccacatccattctcgagcttagcaaacgatgaactccatagtgcctacagttctgaaataaatgcaaattgttatttttgaaccatgtttggaacg -3’ | F: 5’- gaaaattatcgaaaaactgtcacaaac -3’; R: 5’- gttttccattttcaattgaattgacttc -3’;  WT, 563bp; Mutant, 573bp=297bp+276bp (XhoI digestion) |
| *htp-3* null mutant in *ie51* or *ie52* | 5’- agtgccgaaatcggatacgg -3’ and 5’- tgttcatcgacttcaactgg -3’; 5’- gtcactaaacacgaatcccgtggaaacgtttgtctggacctttgtctatgactcaaccacatctgcaagtgccgaaatctgatgaagtcgatgaacagccagaagaacaaaatgatgatgatgctcagaattctcttcaaattgactctgatgctcagaattctcttcaaattgactctg -3’ | N/A |
| *dsb-2::AID::3xFLAG* in *ie58* | 5’- gaatcgtgttgctcaagctg -3’; 5’- gaagatgtgagaatatatttgtctaaaaacaatggaaagaatgtaattttccaggtcaccgaaccaccaataaacgatatattctttgatcgttttcatcaaccacaactcgaacagcacgattctgttccagaagatggagctggatcacctaaagatccagccaaacctccggccaaggcacaagttgtgggatggccaccggtgagatcataccggaagaacgtgatggtttcctgccaaaaatcaagcggtggcccggaggcggcggcgttcgtgaagggagccggatctgattataaagaccatgatggagactataaggatcacgatattgattacaaagacgatgatgataaatgattatttggaaaaattctaaaaatttaaacttgtatcatttgaatctgtaaattttttttcaagtatatttaag -3’ | F: 5’- ctaagcgttgaagtagccgacaaatg -3’; R: 5’- cgagctaaactggtccgcgaagag -3’;  WT, 480bp; inserted, 702bp |
| *spo-11::AID::3xFLAG* in *ie59* or *ie60* | 5’- tactcgtttataatcgtctc -3’; 5’- gacaggagtatatggctcgattcttgattgctccgagagtcatgtcgatagaaaaagaaattccgattcaaccagaaaccatcatcaacgaatacggatcacctaaagatccagccaaacctccggccaaggcacaagttgtgggatggccaccggtgagatcataccggaagaacgtgatggtttcctgccaaaaatcaagcggtggcccggaggcggcggcgttcgtgaagggagccggttctgattataaagaccatgatggagactataaggatcacgatattgattacaaagacgatgatgataaaggtagttcgacagatgatgatcgggaagcaaaagacgatgactatatagattcggatgctgaagaaaagttccagaatatgatagataatgatagtg -3’ | F: 5’- gtgacacgagaattagactggatg -3’; R: 5’- attgaggaggagcactctg -3’;  WT, 532bp; inserted, 730bp |
| *zhp-1::AID::3xFLAG* in *ie61* or *ie62* | 5’- tcgtctcaatcgaatcgtgg -3’; 5’-cgttgctggttcagatgtagataatgacgaattgctcgattatgtatgctgatatcattgatttttctgttcctaaagttgctttttcaggatcttctaggattgaggaatcgatctgattcatcatcatccaactgctcgtctcaatctaacagaggaggatctctgtttggagctggatcacctaaagatccagccaaacctccggccaaggcacaagttgtgggatggccaccggtgagatcataccggaagaacgtgatggtttcctgccaaaaatcaagcggtggcccggaggcggcggcgttcgtgaagggagccggatctgattataaagaccatgatggagactataaggatcacgatattgattacaaagacgatgatgataaataacataatctgtaatatagatgaccatagttttatttttcagctataaacataaaaatgcttgatttatcttcttcccacatatccagagcaaaacgtgacac -3’ | F: 5’- cttcggaatttgtatgtattccctgatcg -3’; R: 5’- cgtgtcgcttgatttatctagtcctag -3’;  WT, 562bp; inserted, 784bp |
| *zhp-2::HA* in *ie63, ie72, ie73, ie74, ie75, ie79, ie84* or *ie107* | 5’- gagttttattgacgaagaat -3’; 5’- gatcataggaatagattgtttggcgcaggacttgagcatccgtcgccaattcttcgtcaaggagccggatcttacccctacgatgtcccagattatgcttaaaactccctgtatgtcacctccgtatttttatcctttttccacaatttttatttc -3’ | F: 5’- gcaattgctgtgattgattaagtg -3’; R: 5’- ggagatattacgggggcaataaac -3’;  WT, 211bp; inserted, 250bp |
| *zhp-3::V5* in *ie64, ie69, ie85, ie92, ie103* or *ie104* | 5’- gagattaaaacattaatcgg -3’; 5’- gttttcaggatacctcgcacaaaggaaaccgatcaatggtcggagcttcattggacccgccgatggagccggatctggaaagccaattccaaacccacttcttggactcgactccacctaatgttttaatctcgtttttttctgaattcgttctttatttgttgatatatacatc -3’ | F: 5’- cgaccctctcatcaactaacac -3’; R: 5’- tttcagcactcttcgggtc -3’;  WT, 434bp; inserted, 488bp |
| *zhp-4::HA* in *ie65, ie70, ie81, ie86, ie87, ie88, ie89* or *ie90* | 5’- gagaaaagcacaaggagcat -3’; 5’- ctctgcaatattttacatgataaaatgcgacacatatattaaaaaaaatcgattcaagcataatctgggacatcgtaggggtatgatccagctccttgactattacgatgctccttgtgcttttctctttgagcttcatgttttttcaagtacgggaac -3’ | F: 5’- ggaaatttatcgattttttcggaaattgattg -3’; R: 5’- catgataaaatgcgacacatatattaaa -3’;  WT, 236bp; inserted, 275bp |
| *zhp-2::AID::3xFLAG* in *ie66* or *ie67* | 5’- gagttttattgacgaagaat -3’; 5’- gcaattgctgtgattgattaagtgtaattttttccagatcgattatagtcgccgtgatcataggaatagattgtttggcgcaggacttgagcatccgtcgccaattcttcgtcaaggagctggatcacctaaagatccagccaaacctccggccaaggcacaagttgtgggatggccaccggtgagatcataccggaagaacgtgatggtttcctgccaaaaatcaagcggtggcccggaggcggcggcgttcgtgaagggagccggatctgattataaagaccatgatggagactataaggatcacgatattgattacaaagacgatgatgataaataaaactccctgtatgtcacctccgtatttttatcctttttccacaatttttatttctattcaaaatcaaaggtttattgcccccgtaatatctcc -3’ | F: 5’- ctccaatgttagttttatctcctgttttc -3’; R: 5’- cgtcgcgttttgtaccataaaacaatc -3’;  WT, 476bp; inserted, 698bp |
| *zhp-1::V5* in *ie68, ie78* or *ie83* | 5’- tcgtctcaatcgaatcgtgg -3’; 5’- catttttatgtttatagctgaaaaataaaactatggtcatctatattacagattatgttaggtggagtcgagtccaagaagtgggtttggaattggctttcctgatccagctccaaacagagatcctcctctgttagattgagacgagcagttggatgatgatgaatcagatcgattcctcaatcctagaagatcc -3’ | F: 5’- cgttgctggttcagatgtagataatg -3’; R: 5’- gtgtcacgttttgctctggatatgtg -3’;  WT, 275bp; inserted, 329bp |
| *zhp-3::AID::3xFLAG* in *ie76* or *ie77* | 5’- gagattaaaacattaatcgg -3’; 5’- gtgtactctgacgttttcaggatacctcgcacaaaggaaaccgatcaatggtcggagcttcattggacccgccgatggagctggatcacctaaagatccagccaaacctccggccaaggcacaagttgtgggatggccaccggtgagatcataccggaagaacgtgatggtttcctgccaaaaatcaagcggtggcccggaggcggcggcgttcgtgaagggagccggatctgattataaagaccatgatggagactataaggatcacgatattgattacaaagacgatgatgataaataatgttttaatctcgtttttttctgaattcgttctttatttgttgatatatacatcatgtacaattcaactttaatg -3’ | F: 5’- cgaccctctcatcaactaacac -3’; R: 5’- tttcagcactcttcgggtc -3’;  WT, 434bp; inserted, 656bp |
| *zhp-3::AID::GFP* in *ie80* | 5’- gagattaaaacattaatcgg-3’; 5’- aactaacacacttacacacttttttcgtctctcactctctcattccgggcgctttttgcgcctttccgctccctctatattccaattctttcgatttttgccacgtgtactctgacgttttcaggatacctcgcacaaaggaaaccgatcaatggtcggagcttcattggacccgccgatggaggatctggaggcggttctggcggaggttctggtcctaaagatccagccaaacctccggccaaggcacaagttgtgggatggccaccggtgagatcataccggaagaacgtgatggtttcctgccaaaaatcaagcggtggcccggaggcggcggcgttcgtgaaggtttctaaaggagaagaacttttcacaggagttgttccaatacttgttgaacttgatggagatgtaaatggacataagttctctgtttctggcgaaggagaaggagatgctacttatggaaaacttactctcaagttcatttgcactactggaaaacttcccgttccatggccaactcttgttactactttgacttatggagttcaatgtttcgctcgttatccagaccatatgaaacaacacgatttcttcaaatctgctatgccagaaggatatgttcaagaaagaactattttcttcaaagatgatggaaactacaaaactcgtgcggaagtaaagtttgagggagatactcttgtaaatagaatagaactcaaaggaatagatttcaaagaagatggaaatatacttggacataaacttgaatataactacaatagtcataaagtttatattactgctgataaacaaaaaaatggaataaaagtaaacttcaaaactcgtcataatatagaagatggaagtgttcaacttgctgaccattaccaacaaaatactccaataggagatggcccagttcttctcccagataatcattatctttctactcaatctgctctttctaaagacccaaatgaaaaaagagaccatatggttcttttagagttcgttactgcggcgggaataactcttggaatggatgaactttacaaataatgttttaatctcgtttttttctgaattcgttctttatttgttgatatatacatcatgtacaattcaactttaatgtttatttcataaaaattgggttaaaaattccaaatttctttttctctacctagttcacatctgatttaaattttatttgaatatttcg -3’ | F: 5’- cgaccctctcatcaactaacac -3’; R: 5’- cacgttcttccggtatgatctc -3’;  WT, no product; inserted, 307bp.  or  F: 5’- cgaccctctcatcaactaacac -3’; R2: 5’- tttcagcactcttcgggtc -3’  WT, 434bp; inserted, 1316bp |
| *zhp-4::AID::3xFLAG* in *ie82* | 5’- gagaaaagcacaaggagcat -3’; 5’- gttcccgtacttgaaaaaacatgaagctcaaagagaaaagcacaaggagcatcgtaatagtcaaggagctggatcacctaaagatccagccaaacctccggccaaggcacaagttgtgggatggccaccggtgagatcataccggaagaacgtgatggtttcctgccaaaaatcaagcggtggcccggaggcggcggcgttcgtgaagggagccggatctgattataaagaccatgatggagactataaggatcacgatattgattacaaagacgatgatgataaatgaatcgattttttttaatatatgtgtcgcattttatcatg -3’ | F: 5’- ggaaatttatcgattttttcggaaattgattg -3’; R: 5’- ctaaaactgtactttcaggtcataaatgtatc -3’;  WT, 382bp; inserted, 604bp |
| *plk-2::HA* in *ie105, ie106, ie109* or *ie110* | 5’- tcgattttcttagcgacgcg -3’; 5’-caacgtccgtttggaatctgcagcagatatccagccggcttatccatcatcctcgcgtcgcggagccggatcttacccctacgatgtcccagattatgcttaagaaaatcgatctgcaacaaattgagctcatttccccttaccggttttg -3’ | F: 5’- ggagaagttcctccatcgaattc -3’; R: 5’- ggataaaattgattacaaacacggac -3’;  WT, 294bp; inserted, 333bp |
| *plk-2::AID::3xFLAG* in *ie108* | 5’- tcgattttcttagcgacgcg -3’; 5’-gaaagaggagacgaaacacaatgcaccggcggccaatgcagtacgccttccatcgacttccagcaacgtccgtttggaatctgcagcagatatccagccggcttatccatcatcctcgcgtcgcggagctggatcacctaaagatccagccaaacctccggccaaggcacaagttgtgggatggccaccggtgagatcataccggaagaacgtgatggtttcctgccaaaaatcaagcggtggcccggaggcggcggcgttcgtgaagggagccggatctgattataaagaccatgatggagactataaggatcacgatattgattacaaagacgatgatgataaataagaaaatcgatctgcaacaaattgagctcatttccccttaccggttttgatatttctctgatcaatacacttttatgtccgtgtttgtaatcaattttatcc -3’ | F: 5’- ggagaagttcctccatcgaattc -3’; R: 5’- gagcatgatgacacccgaatgtttg -3’;  WT, 451bp; inserted, 673bp |

**Table S4**

**Strains used in this study**

| Strains | Source | Identifier |
| --- | --- | --- |
| *C. elegans:* Hawaiian wild-type isolate | Caenorhabditis Genetics Center | CB4856 |
| *C. elegans: syp-1 (me17) V/nT1[unc-?(n754) let-? qIs50] (IV;V)* | MacQueen et al., 2002; Caenorhabditis Genetics Center | AV307/CA30 |
| *C. elegans: spo-11(me44) IV/ieT1* | Chan et al., 2004; Hayashi et al., 2007; this paper | AV157 |
| *C. elegans: dsb-2(tm6047) II* | This paper; 421 bp deletion  null allele mutant; [Japanese KO consortium](http://shigen.nig.ac.jp/c.elegans/mutants/DetailsSearch?lang=english&seq=6047) | CA1153 |
| *C. elegans: zhp-3(jf61) I/hT2 [bli-4(e937) let-?(q782) qIs48] (I; III)* | Jantsch et al., 2004; Bhalla et al., 2008 | UV1/CA685 |
| *C. elegans: msh-5(me23) IV/nT1 [unc-?(n754) let-?] (IV;V)* | Kelly et al., 2000; Caenorhabditis Genetics Center | AV115 |
| *C. elegans: meIs8[pie-1p::GFP::cosa-1, unc-119(+)] II* | Yokoo et al., 2012; Caenorhabditis Genetics Center | AV630 |
| *C. elegans: ieSi38[sun-1p::TIR1::mRuby::sun-1 3’ UTR, Cbr-unc-119 (+)] IV* | Zhang et al., 2015; Caenorhabditis Genetics Center | CA1199 |
| *C. elegans: zhp-1(ie43) I/ hT2 [bli-4(e937) let-?(q782) qIs48] (I; III)* | This paper | CA1356 |
| *C. elegans: zhp-1(ie44) I/ hT2 [bli-4(e937) let-?(q782) qIs48] (I; III)* | This paper | CA1357 |
| *C. elegans: zhp-2(ie45) I/ hT2 [bli-4(e937) let-?(q782) qIs48] (I; III)* | This paper | CA1358 |
| *C. elegans: zhp-1(ie46) I in Hawaii background* | This paper | CA1359 |
| *C. elegans: meIs8[pie-1p::GFP::cosa-1, unc-119(+)] II; ieSi38[sun-1p::TIR1::mRuby::sun-1 3'UTR, Cbr-unc-119(+)] IV* | This paper | CA1364 |
| *C. elegans: zhp-1(ie61[zhp-1::AID::3xFLAG]) I; unc-119(ed3) III; ieSi38[sun-1p::TIR1::mRuby::sun-1 3'UTR, Cbr-unc-119(+)] IV* | This paper | CA1366 |
| *C. elegans: zhp-1(ie62[zhp-1::AID::3xFLAG]) I; meIs8[pie-1p::GFP::cosa-1, unc-119(+)] II; ieSi38[sun-1p::TIR1::mRuby::sun-1 3'UTR, Cbr-unc-119(+)] IV* | This paper | CA1367 |
| *C. elegans: htp-3(ie51), zhp-1(ie62[zhp-1::AID::3xFLAG]) I; meIs8[pie-1p::GFP::cosa-1, unc-119(+)] II; ieSi38[sun-1p::TIR1::mRuby::sun-1 3'UTR, Cbr-unc-119(+)] IV* | This paper | CA1368 |
| *C. elegans: zhp-1(ie62[zhp-1::AID::3xFLAG]) I; meIs8[pie-1p::GFP::cosa-1, unc-119(+)] II; ieSi38[sun-1p::TIR1::mRuby::sun-1 3'UTR, Cbr-unc-119(+)], spo-11(ie59[spo-11::AID::3xFLAG]) IV* | This paper | CA1369 |
| *C. elegans: zhp-1(ie61[zhp-1::AID::3xFLAG]), zhp-2(ie63[zhp-2::HA]) I; unc-119(ed3) III; ieSi38[sun-1p::TIR1::mRuby::sun-1 3'UTR, Cbr-unc-119(+)] IV;* | This paper | CA1370 |
| *C. elegans: zhp-1(ie61[zhp-1::AID::3xFLAG]), zhp-3(ie64[zhp-3::V5]) I; unc-119(ed3) III; ieSi38[sun-1p::TIR1::mRuby::sun-1 3'UTR, Cbr-unc-119(+)] IV;* | This paper | CA1371 |
| *C. elegans: zhp-1(ie61[zhp-1::AID::3xFLAG]) I; unc-119(ed3) III; ieSi38[sun-1p::TIR1::mRuby::sun-1 3'UTR, Cbr-unc-119(+)] IV; zhp-4(ie65[zhp-4::HA]) V* | This paper | CA1372 |
| *C. elegans: zhp-2(ie66[zhp-2::AID::3xFLAG]) I; unc-119(ed3) III; ieSi38[sun-1p::TIR1::mRuby::sun-1 3'UTR, Cbr-unc-119(+)] IV* | This paper | CA1374 |
| *C. elegans: zhp-2(ie67[zhp-2::AID::3xFLAG]) I; meIs8[pie-1p::GFP::cosa-1, unc-119(+)] II; ieSi38[sun-1p::TIR1::mRuby::sun-1 3'UTR, Cbr-unc-119(+)] IV* | This paper | CA1375 |
| *C. elegans: htp-3(ie52), zhp-2(ie66[zhp-2::AID::3xFLAG]) I; meIs8[pie-1p::GFP::cosa-1, unc-119(+)] II; ieSi38[sun-1p::TIR1::mRuby::sun-1 3'UTR, Cbr-unc-119(+)] IV* | This paper | CA1376 |
| *C. elegans: zhp-2(ie66[zhp-2::AID::3xFLAG]) I; meIs8[pie-1p::GFP::cosa-1, unc-119(+)] II; ieSi38[sun-1p::TIR1::mRuby::sun-1 3'UTR, Cbr-unc-119(+)], spo-11(ie60[spo-11::AID::3xFLAG]) IV* | This paper | CA1377 |
| *C. elegans: zhp-1(ie68[zhp-1::V5]), zhp-2(ie66[zhp-2::AID::3xFLAG]) I; unc-119(ed3) III; ieSi38[sun-1p::TIR1::mRuby::sun-1 3'UTR, Cbr-unc-119(+)] IV* | This paper | CA1378 |
| *C. elegans: zhp-2(ie66[zhp-2::AID::3xFLAG]), zhp-3(ie69[zhp-3::V5]) I; unc-119(ed3) III; ieSi38[sun-1p::TIR1::mRuby::sun-1 3'UTR, Cbr-unc-119(+)] IV* | This paper | CA1379 |
| *C. elegans: zhp-2(ie66[zhp-2::AID::3xFLAG]) I; unc-119(ed3) III; ieSi38[sun-1p::TIR1::mRuby::sun-1 3'UTR, Cbr-unc-119(+)] IV; zhp-4(ie70[zhp-4::HA]) V* | This paper | CA1380 |
| *C. elegans: zhp-2(ie72[zhp-2::HA]) I; syp-1(me17) V/nT1[unc-?(n754) let-? qIs50] (IV;V)* | This paper | CA1383 |
| *C. elegans: zhp-2(ie73[zhp-2::HA]) I; spo-11(me44) IV/ ieT1* | This paper | CA1384 |
| *C. elegans: zhp-2(ie74[zhp-2::HA]) I; msh-5(me23) IV/nT1[unc-?(n754) let-?] (IV;V)* | This paper | CA1385 |
| *C. elegans: zhp-2(ie75[zhp-2::HA]) I; cosa-1(we12) III/qC1* | This paper | CA1386 |
| *C. elegans: zhp-3(ie76[zhp-3::AID::3xFLAG]) I; unc-119(ed3) III; ieSi38[sun-1p::TIR1::mRuby::sun-1 3'UTR, Cbr-unc-119(+)] IV* | This paper | CA1387 |
| *C. elegans: zhp-3(ie77[zhp-3::AID::3xFLAG]) I; meIs8[pie-1p::GFP::cosa-1, unc-119(+)] II; ieSi38[sun-1p::TIR1::mRuby::sun-1 3'UTR, Cbr-unc-119(+)] IV* | This paper | CA1388 |
| *C. elegans: zhp-1(ie78[zhp-1::V5]), zhp-3(ie76[zhp-3::AID::3xFLAG]) I; unc-119(ed3) III; ieSi38[sun-1p::TIR1::mRuby::sun-1 3'UTR, Cbr-unc-119(+)] IV* | This paper | CA1389 |
| *C. elegans: zhp-2(ie79[zhp-2::HA]), zhp-3(ie76[zhp-3::AID::3xFLAG]) I; unc-119(ed3) III; ieSi38[sun-1p::TIR1::mRuby::sun-1 3'UTR, Cbr-unc-119(+)] IV* | This paper | CA1390 |
| *C. elegans: zhp-3(ie80[zhp-3::AID::GFP]) I; unc-119(ed3) III; ieSi38[sun-1p::TIR1::mRuby::sun-1 3'UTR, Cbr-unc-119(+)] IV* | This paper | CA1391 |
| *C. elegans: zhp-3(ie80[zhp-3::AID::GFP]) I; unc-119(ed3) III; ieSi38[sun-1p::TIR1::mRuby::sun-1 3'UTR, Cbr-unc-119(+)] IV; zhp-4(ie81[zhp-4::HA]) V* | This paper | CA1392 |
| *C. elegans: unc-119(ed3) III; ieSi38[sun-1p::TIR1::mRuby::sun-1 3'UTR, Cbr-unc-119(+)] IV; zhp-4(ie82[zhp-4::AID::3xFLAG]) V* | This paper | CA1393 |
| *C. elegans: meIs8[pie-1p::GFP::cosa-1, unc-119(+)] II; ieSi38[sun-1p::TIR1::mRuby::sun-1 3'UTR, Cbr-unc-119(+)] IV; zhp-4(ie82[zhp-4::AID::3xFLAG]) V* | This paper | CA1394 |
| *C. elegans: zhp-1(ie83[zhp-1::V5]) I; unc-119(ed3) III; ieSi38[sun-1p::TIR1::mRuby::sun-1 3'UTR, Cbr-unc-119(+)] IV; zhp-4(ie82[zhp-4::AID::3xFLAG]) V* | This paper | CA1395 |
| *C. elegans: zhp-2(ie84[zhp-2::HA]) I; unc-119(ed3) III; ieSi38[sun-1p::TIR1::mRuby::sun-1 3'UTR, Cbr-unc-119(+)] IV; zhp-4(ie82[zhp-4::AID::3xFLAG]) V* | This paper | CA1396 |
| *C. elegans: zhp-3(ie85[zhp-3::V5]) I; unc-119(ed3) III; ieSi38[sun-1p::TIR1::mRuby::sun-1 3'UTR, Cbr-unc-119(+)] IV; zhp-4(ie82[zhp-4::AID::3xFLAG]) V* | This paper | CA1397 |
| *C. elegans: zhp-3(jf61) I/ hT2 [bli-4(e937) let-?(q782) qIs48] (I; III); zhp-4(ie86[zhp-4::HA]) V* | This paper | CA1398 |
| *C. elegans: syp-1(me17) V/nT1[unc-?(n754) let-? qIs50] (IV;V); zhp-4(ie87[zhp-4::HA]) V* | This paper | CA1399 |
| *C. elegans: spo-11(me44) IV/ieT1; zhp-4(ie88[zhp-4::HA]) V* | This paper | CA1400 |
| *C. elegans: msh-5(me23) IV/nT1[unc-?(n754) let-?] (IV;V); zhp-4(ie89[zhp-4::HA]) V* | This paper | CA1401 |
| *C. elegans: cosa-1(we12) III/qC1; zhp-4(ie90[zhp-4::HA]) V* | This paper | CA1402 |
| *C. elegans: zhp-3(ie92[zhp-3::V5]) I* | This paper | CA1404 |
| *C. elegans: zhp-3(ie92[zhp-3::V5]) I; dsb-2(tm6047) II* | This paper | CA1405 |
| *C. elegans: zhp-1(ie78[zhp-1::V5]), zhp-3(ie76[zhp-3::AID::3xFLAG]) I; syp-1 (me17) V/nT1[unc-?(n754) let-? qIs50] (IV;V)* | This paper | CA1410 |
| *C. elegans: dsb-2(ie58[dsb-2::AID::3xFLAG]), meIs8[pie-1p::GFP::cosa-1, unc-119(+)] II; ieSi38[sun-1p::TIR1::mRuby::sun-1 3'UTR, Cbr-unc-119(+)] IV* | This paper | CA1421 |
| *C. elegans: zhp-2(ie67[zhp-2::AID::3xFLAG]) I; dsb-2(ie58[dsb-2::AID::3xFLAG]), meIs8[pie-1p::GFP::cosa-1, unc-119(+)] II; ieSi38[sun-1p::TIR1::mRuby::sun-1 3'UTR, Cbr-unc-119(+)] IV* | This paper | CA1422 |
| *C. elegans: meIs8[pie-1p::GFP::cosa-1, unc-119(+)] II; ieSi38[sun-1p::TIR1::mRuby::sun-1 3'UTR, Cbr-unc-119(+)], spo-11(ie59[spo-11::AID::3xFLAG]), IV* | This paper | CA1423 |
| *C. elegans: zhp-1(ie62[zhp-1::AID::3xFLAG]) I; meIs8[pie-1p::GFP::cosa-1, unc-119(+)] II; ieSi38[sun-1p::TIR1::mRuby::sun-1 3'UTR, Cbr-unc-119(+)] IV; zhp-4(ie82[zhp-4::AID::3xFALG]) V* | This paper | CA1424 |
| *C. elegans: zhp-3(ie103[zhp-3::V5]) I; dsb-2(ie58[dsb-2::AID::3xFLAG]), meIs8[pie-1p::GFP::cosa-1, unc-119(+)] II; ieSi38[sun-1p::TIR1::mRuby::sun-1 3'UTR, Cbr-unc-119(+)] IV* | This paper | CA1425 |
| *C. elegans: zhp-2(ie67[zhp-2::AID::3xFLAG]), zhp-3(ie104[zhp-3::V5]) I; dsb-2(ie58[dsb-2::AID::3xFLAG]), meIs8[pie-1p::GFP::cosa-1, unc-119(+)] II; ieSi38[sun-1p::TIR1::mRuby::sun-1 3'UTR, Cbr-unc-119(+)] IV* | This paper | CA1426 |
| *C. elegans: plk-2(ie105[plk-2::HA]), zhp-3(ie103[zhp-3::V5]) I; dsb-2(ie58[dsb-2::AID::3xFLAG]), meIs8[pie-1p::GFP::cosa-1, unc-119(+)] II; ieSi38[sun-1p::TIR1::mRuby::sun-1 3'UTR, Cbr-unc-119(+)] IV* | This paper | CA1427 |
| *C. elegans: plk-2(ie106[plk-2::HA]), zhp-2(ie67[zhp-2::AID::3xFLAG]), zhp-3(ie104[zhp-3::V5]) I; dsb-2(ie58[dsb-2::AID::3xFLAG]), meIs8[pie-1p::GFP::cosa-1, unc-119(+)] II; ieSi38[sun-1p::TIR1::mRuby::sun-1 3'UTR, Cbr-unc-119(+)] IV* | This paper | CA1428 |
| *C. elegans: plk-2(ie108[plk-2::AID::3xFLAG]), zhp-2(ie107[zhp-2::HA]) I; meIs8[pie-1p::GFP::cosa-1, unc-119(+)] II; ieSi38[sun-1p::TIR1::mRuby::sun-1 3'UTR, Cbr-unc-119(+)] IV* | This paper | CA1429 |
| *C. elegans: plk-2(ie109[plk-2::HA]), zhp-1(ie62[zhp-1::AID::3xFLAG]) I; meIs8[pie-1p::GFP::cosa-1, unc-119(+)] II; ieSi38[sun-1p::TIR1::mRuby::sun-1 3'UTR, Cbr-unc-119(+)] IV* | This paper | CA1430 |
| *C. elegans: plk-2(ie110[plk-2::HA]), zhp-2(ie67[zhp-1::AID::3xFLAG]) I; meIs8[pie-1p::GFP::cosa-1, unc-119(+)] II; ieSi38[sun-1p::TIR1::mRuby::sun-1 3'UTR, Cbr-unc-119(+)] IV* | This paper | CA1431 |
